# Heart Rate Variability in Children with Tricyclic Antidepressant Intoxication

**DOI:** 10.1155/2013/196506

**Published:** 2013-03-05

**Authors:** Ener Cagri Dinleyici, Zubeyir Kilic, Sabiha Sahin, Rabia Tutuncu-Toker, Makbule Eren, Zeynel Abidin Yargic, Pelin Kosger, Birsen Ucar

**Affiliations:** ^1^Department of Pediatric Intensive Care, Faculty of Medicine, Eskişehir Osmangazi University, 26480 Eskişehir, Turkey; ^2^Pediatric Intensive Care Unit, Faculty of Medicine, Eskişehir Osmangazi University, 26480 Eskişehir, Turkey; ^3^Department of Pediatric Cardiology, Faculty of Medicine, Eskişehir Osmangazi University, 26480 Eskişehir, Turkey; ^4^Department of Pediatrics, Faculty of Medicine, Eskişehir Osmangazi University, 26480 Eskişehir, Turkey

## Abstract

The aim of this study was to evaluate HRV in children requiring intensive care unit stays due to TCA poisoning between March 2009 and July 2010. In the time-domain nonspectral evaluation, the SDNN (*P* < 0.001), SDNNi (*P* < 0.05), RMSDD (*P* < 0.01), and pNN50 (*P* < 0.01) were found to be significantly lower in the TCA intoxication group. The spectral analysis of the data recorded during the first 5 minutes after intensive care unit admission showed that the values of the nLF (*P* < 0.05) and the LF/HF ratio (*P* = 0.001) were significantly higher in the TCA intoxication group, while the nHF (*P* = 0.001) values were significantly lower. The frequency-domain spectral analysis of the data recorded during the last 5 minutes showed a lower nHF (*P* = 0.001) in the TCA intoxication group than in the controls, and the LF/HF ratio was significantly higher (*P* < 0.05) in the intoxication group. The LF/HF ratio was higher in the seven children with seizures (*P* < 0.001). These findings provided us with a starting point for the value of HRV analysis in determining the risk of arrhythmia and convulsion in TCA poisoning patients. HRV can be used as a noninvasive testing method in determining the treatment and prognosis of TCA poisoning patients.

## 1. Introduction

Poisoning is a common and important cause of morbidity and mortality worldwide, especially during childhood. More than 50% of patients reported to poison control centers are adolescents or less than five years of age [1]. Among the substances that cause poisoning, drugs have been reported as the most common factor. In Turkey, tricyclic antidepressants (TCA) occupy an important place among the causes of poisoning, and the incidence of such events has increased over the last decade [2]. TCAs (amitriptyline, nortriptyline, clomipramine, desipramine, imipramine, doxepinm, and protriptyline) are frequently used for the treatment of depression, chronic pain syndrome, school phobia, hyperkinesias, nocturnal enuresis, and attention deficit-hyperactivity disorder [3]. 

TCA-related poisoning influences the autonomic nervous system, central nervous system (CNS), and cardiovascular system (CVS), and clinical signs appear within 6–8 hours. There is no laboratory test to precisely diagnose TCA-related poisoning, and it is impossible to predict the prognosis. The semiquantitative enzyme immunoassay technique may be useful for measuring TCA levels; however, there is no correlation between drug levels and clinical findings [4]. The most severe life-threatening condition in TCA-related poisoning is the dysrhythmias that occur due to the quinidine-like effects on the myocardial tissue. Electrocardiogram (ECG) findings vary [5], and Boehnert and Lovejoy [6] have shown an association between a QRS duration >100 ms and the risk of arrhythmia or seizures. 

Heart rate variability (HRV) can be described as instantaneous differences in sinus rate over time or heart rate fluctuations in the mean heart rate [7, 8]. Heart rate variability, which can be analyzed using ECG recordings, is a noninvasive method to assess cardiac autonomic activity. Changes can occur in heart rate that are related to autonomic tone due to exercise, physical and mental stress, and respiratory and metabolic reasons. A close relationship between increased sympathetic activity or decreased parasympathetic activity and a tendency to fatal arrhythmia has been demonstrated [7]. HRV analysis that reflects the sympathetic and parasympathetic balance is used as a measure of cardiac autonomic tone. Generally, 24-hour-long sequences should be obtained to get reliable results for time-domain measures, and short-term recordings are needed to analyze the frequency-domain parameters. Studies regarding HRV in children are limited compared with the number of adult studies. HRV analysis seems to be promising in the study of the cardiovascular responses to autonomic tone changes in obese children [9, 10]. HRV analyses have been studied in children with beta-thalassemia [11, 12], epilepsy [13], recurrent neurocardiogenic syncope [14], and obstructive sleep apnea [15, 16].

HRV plays an important role in describing fatal or near-fatal arrhythmias [17] because increases in sympathetic activity can cause severe arrhythmias and sudden death [18]. Recently, a meta-analysis showed that the therapeutic use of TCAs caused a considerable decrease in HRV [19]. There have been analyses showing HRV changes due to TCA-related poisoning in adults, and differences in HRV have been suggested to be predictors for ventricular arrhythmias in an adult patient [20, 21]. TCAs can be ingested accidentally or voluntarily by children or therapeutic mistakes can occur. To our knowledge, there have been no studies on the effects of TCA-related poisoning on HRV. In the present study, we aimed to determine the effect of TCA poisoning on heart rate variability and to assess the effects of these results on clinical findings.

## 2. Materials and Methods

This prospective study was planned to involve children with isolated cases of TCA intoxication who had been referred to the Pediatric Emergency Department of Eskisehir Osmangazi University. We planned to enroll 20 children because our previous records showed that approximately 20 children had been admitted to our clinic for TCA poisoning during the last two years. For allocated randomization, 20 age- and sex-matched healthy children were included as a control group. The children in the control group had no underlying diseases and were admitted to our clinic for routine checkups. Holter analyses of the control children were recorded in their homes to avoid hospital stress. Essential equipment for the HRV analysis was provided within the scope of the Eskisehir Osmangazi University Scientific Research Project (project number: 200811022). The study protocol was approved by the local Ethical Committee of Eskisehir Osmangazi University, and the parents gave informed consent for the study.

TCA intoxication was diagnosed at >300 ng/mL of TCA as measured using semiquantitative enzyme immunoassay method in children who were referred to our clinic with a history or clinical findings of poisoning. The exclusion criteria were a history of drug intake apart from TCA, a different drug toxicity according to the laboratory measurements, the presence of chronic underlying diseases before poisoning, and the presence of obesity according to anthropometric measurements. The clinical findings for the patients who were included in the study were recorded during their PICU stay. Standard 12-lead electrocardiography and ambulatory blood pressure measurements were also recorded. To perform the HRV analysis, HRV recordings were scheduled during the first half an hour after PICU admission in all of the cases. Recordings for a period of 24 hours that included the values for an entire day and night were completed for both the study and control groups. The parameters of the HRV analysis were calculated in compliance with the recommendations of the American Heart Association and the European Society of Cardiology, and the following parameters were evaluated in the HRV analysis [7].

### 2.1. Time-Domain (Nonspectral) Analysis

This analysis was based on the assessment of the intervals between normal beats on 24-hour ECG recordings. During the statistical analysis, generally all of the QRS complexes, the duration between consecutive QRS complexes (NN interval), or the instantaneous heart rates during continuous ECG recordings are determined [7]. We recorded the following time-domain indices for our HRV analysis: the standard deviation of all of the normal sinus RR intervals over 24 hours (ms) (SDNN), the percentage of successive normal sinus RR intervals exceeding 50 ms (%) (pNN50), and the root mean square of the successive normal sinus RR interval differences (ms) (rMSSD). The SDANN (ms) and SDNN indices (ms) were also recorded in all of the studied children. 

### 2.2. Frequency-Domain (Spectral) Measures

Frequency-domain measures give information about how the power is distributed as a function of frequency. We planned to use parametrical methods. The advantages of parametrical methods are as follows: smoother spectral components that can be distinguished as independent from preselected frequency bands, easy postprocessing of the spectrum with an automatic calculation of low- and high-frequency power components and an easy identification of the central frequency of each component, and accurate estimation even on a small number of samples. We analyzed two different frequency domain calculations during the first and last 5 minutes of the 24-hour recordings in both the patient and control groups. The frequency domain indices were the low-frequency component (LF) (frequency range: 0.04–0.15 Hz), the high-frequency component (HF) (frequency range: 0.15–0.40 Hz), and the very low frequency component (VLF) (frequency range: 0.003–0.04 Hz) [7, 8]. Three values were noted for the first and last 5-minute recordings. A normalized unit (nu) represented the relative value of the LF or HF power components in proportion to the total power minus the VLF power. The normalization minimized the effect of changes in total power on the values of LF and HF. The unit of the power components of the VLF, LF, and HF was ms^2^, but the unit of the normalized LF (nLF) and normalized HF (nHF) was a normalized unit (nu). We used the nLF and nHF in the statistics. In addition, the LF/HF ratio was used to evaluate the balance between the sympathetic and parasympathetic activities [7, 8].

Statistical analysis of study data was performed using SPSS 16.0 Software (Chicago, IL). The *Mann-Whitney U* test was used for comparisons. Comparisons over time were performed using the *Wilcoxon* test, and correlations were evaluated using the Spearman correlation test. A *P* < 0.05 was considered statistically significant.

## 3. Results

Twenty children (nine boys and eleven girls) between the ages of 3 and 16 years old following admittance to the PICU with TCA intoxication (TCA level > 300 ng/mL) and 20 healthy children (10 boys and 10 girls) were included in the study between March 2009 and July 2010. Sixteen of the patients presenting with TCA intoxication had ingested the drug for the purpose of suicide, and 4 had ingested the drug accidently. The median time to arrive in the emergency department was 2.4 hours (range 0.5–4 hours). The total serum TCA level ranged from 380 to 2000 ng/mL (mean 1116 ± 635 ng/mL). The most commonly observed clinical sign was the loss of consciousness (*n* = 16, 80%). Seizure was observed in 7 cases (35%). The most frequent cardiovascular sign was sinus tachycardia (85%, *n* = 17). The mean heart rate was 148 ± 36 beats/minute (range 66–224 beats/min). The mean systolic blood pressure was 115 mmHg (range 62–160 mmHg), and the mean diastolic blood pressure was 58 mmHg (range 36–92 mmHg) during the blood pressure monitoring. The mean systolic and diastolic blood pressures were normal in the control group. Although there were some differences in blood pressure in the TCA intoxication group, no statistical difference was found between the groups (*P* > 0.05). Abnormal ECG results were found in 12 of the cases (60%). QTc prolongation was observed in 5 cases, PR interval prolongation was observed in 3 cases, and QRS interval prolongation was observed in 4 cases (Table 1). ST-T alteration was observed in only one case. No Brugada sign was observed. There was a positive correlation between serum TCA levels and the QRS interval (*r* = 0.684; *P* < 0.01). The mean age was higher in patients with positive ECG findings compared with patients with normal ECG results (median 14.6 years versus 11.2 years, resp., *P* < 0.05). The TCA levels in patients with positive ECG findings were significantly higher than in patients without positive ECG findings (1523 ± 571 ng/mL versus 619 ± 211 ng/mL; *P* = 0.001). The duration of the PICU stay was markedly longer in patients with positive ECG findings compared with patients without ECG findings (median 5.4 days versus 2.4 days; *P* < 0.01). All of the cases were treated using activated charcoal and alkalization. No case was treated using physostigmine, plasmapheresis, or hemoperfusion. One patient was mechanically ventilated for a period of 24 hours. The median duration of the intensive care unit stay was 4.7 days (range 1–9 days), and total duration of hospital stay was 5.4 days (range 3–12 days). All of the patients were successfully treated with these treatments and discharged.

### 3.1. HRV Analysis

The time-domain nonspectral parameters (SDNN, SDANN, RMSDD, SDNN index, NN50, and PNN50) recorded over the course of 24 hours were evaluated in all of the patients and controls, and the results are shown as the median and 95% CI in Table 2. The median SDNN, RMSDD, and SDNN index values were lower in the TCA group than in the controls (*P* < 0.001, *P* < 0.05, and *P* < 0.01, resp.). In the TCA intoxication group, the pNN50 values were lower (*P* < 0.01 for both). The SDNN (*P* < 0.001), RMSDD (*P* < 0.01), SDDNi (*P* < 0.01), and pNN50 (*P* < 0.01) values were lower in the TCA intoxication group among patients with ECG findings compared with patients without positive ECG findings. 

**Figure 1:**
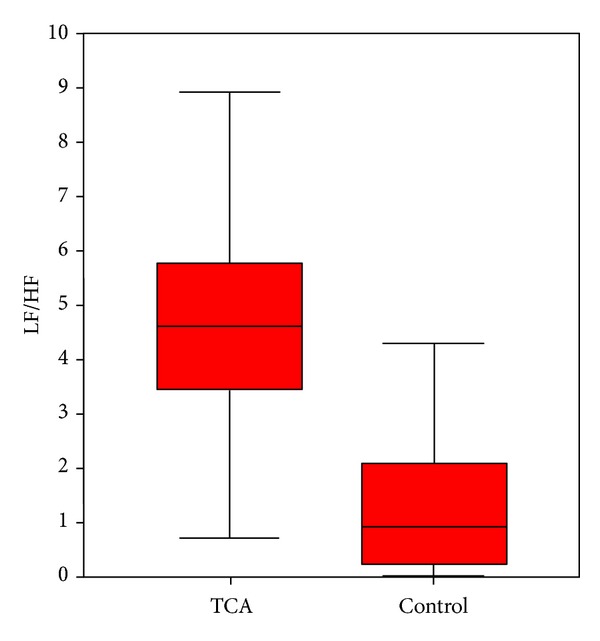
Comparison of the LF/HF ratio within the first 5 minutes between children with TCA intoxication and controls.

There was no significant difference in the VLF values during the first 5 minutes of the intensive care unit stay between the TCA intoxication and control groups (*P* > 0.05). The nLF was markedly higher in the TCA intoxication group compared with the control group (*P* < 0.05), and the nHF was markedly lower in the TCA intoxication group compared with the control group (*P* = 0.001). Also, the LF/HF ratio was calculated to be markedly higher in the TCA intoxication group compared with the control group (*P* = 0.001, Table 2 and Figure 1). 

The frequency-domain spectral analysis was repeated for the last 5 minutes during the 24-hour HRV recordings in both groups. Similarly to the first 5 minutes, the VLF values were found to be similar for both groups. While there was no difference in the nLF values, the nHF was lower in the TCA intoxication group compared with the patients during the first 5 minutes (*P* = 0.001). The LF/HF ratio at the end of the 24 hours in the TCA intoxication group was higher than that in control group (*P* < 0.05).

On comparing the first and last 5-minute recordings of the 20 children with TCA intoxication, no differences were determined for the VLF, nHF, or LF/HF ratio; however, the nLF value for the patients whose nLF value was higher continued to remain high (*P* < 0.05).

**Figure 2:**
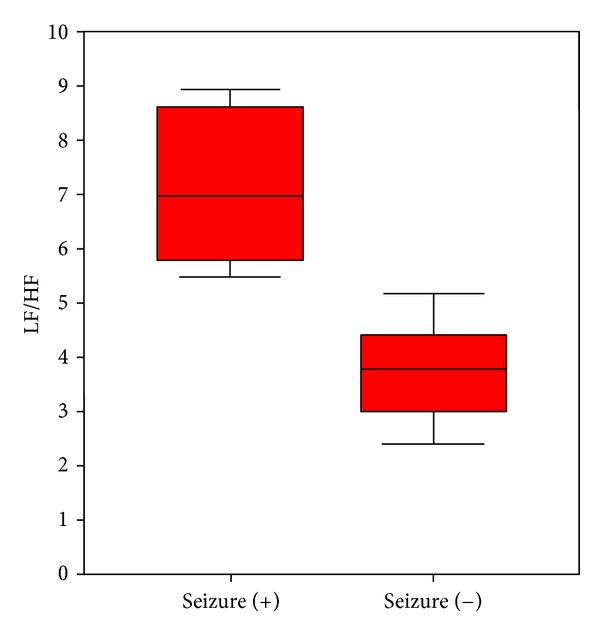
Comparison of the LF/HF ratio during the first 5 minutes of the HRV analysis according to the presence of seizures in patients with TCA intoxication.

There were no significant differences in the time-domain nonspectral analysis parameters between cases with or without seizures; however, the nLF value during the first 5 minutes was higher (though not statistically significantly higher), and the nHF value was lower (*P* < 0.05) in the group who had seizures. Also, the LF/HF ratio was found to be significantly higher in patients who had seizures compared with patients without seizures (median 6.95 versus 3.79, *P* < 0.001) (Figure 2).

When we checked the association between the serum TCA levels and the HRV parameters in the patients with TCA intoxication, we found that the TCA levels showed a negative correlation with the SDNN (*r* = −0.883, *P* < 0.001), RMSDD (*r* = −0.564, *P* < 0.01), SDNNi (*r* = −0.469, *P* < 0.05), SDSD (*r* = −0.564, *P* < 0.01), and pNN50 (*r* = −0.601, *P* < 0.01). We found no significant association between the TCA levels and the spectral analysis parameters (nLF, NHF, LF/HF) during the first and last 5 minutes of recording.

## 4. Discussion

Tricyclic antidepressant intoxication can lead cardiovascular, respiratory, and central nervous system signs. The cardiovascular effects of TCA intoxication include PR or QT interval changes, QRS complex enlargement, and ventricular or supraventricular tachyarrhythmia, which originates basically from the quinidine-like effects of these drugs on heart tissue [22]. The most frequently observed dysrhythmia is sinus tachycardia, which was observed in our study, and wide complex tachycardia (supraventricular or ventricular) remains one of the characteristic complications of TCA intoxication [23]. Leonard et al. [24] similarly have described tachycardia as the most frequent cardiac sign, and they have shown that sinus tachycardia accompanies PR, QRS, and QTc changes in children similarly to adults; however, ECG findings have no diagnostic value for evaluating TCA intoxication in children and adults [23]. It has been concluded that increases >0.10 sec in the QRS interval length, >3 mm in the height of aVR R amplitude, and the abnormalities on the last 40 ms of a QRS axis on the frontal axis are significant for convulsion and ventricular arrhythmia in cases with TCA intoxication [6]. But there is no predictive value for the assessment of the terminal 40 ms in children as in adults [25]. *A* > 0.10 sec QRS duration in extremity derivations has been associated with convulsion, and *A* > 0.16 sec QRS interval has been associated with ventricular arrhythmia. Çitak et al. [26] reported cardiac signs in almost half of all patients with convulsion among their cases. In our study, 7 cases (35%) had seizures; however, no association was found between ECG changes and seizures. There were also no associations between the TCA levels and the presence of conduction disorders or serious cardiac dysrhythmias [27]. In our study, a positive correlation was found between TCA levels and QRS duration, and the serum TCA levels in children with ECG findings were higher in our study. Our results indicated that ECG findings can be used to evaluate TCA intoxication; however, our patient number was not sufficient to show evidence of an association between the ECG findings and serum TCA levels.

HRV analysis has been used to diagnose, follow up, or determine the prognosis of many disorders and is presumed to be safe as a method to quantitatively assess changes in the autonomic nervous system and determine the cardiovascular response to these changes. In many conditions, the balance between the sympathetic and parasympathetic systems is affected, and cardiac autonomic function disorders appear [28, 29]. Abnormalities on HRV as a marker of cardiac autonomic control are presumed to be risk factors for arrhythmia, and it has been shown that they can be used as reliable noninvasive markers of the presence of an underlying disease [30–33]. In our study, HRV abnormalities were clearly shown in children with TCA intoxication during the first 24 hours of their admission. With regard to time-domain analysis, SDNN reflected the overall HRV and the SDSS and RMSSD reflected the vagal tone. In addition, the RMSSD and pNN50 parameters indicated high-frequency variations in heart rate independently from diurnal or other changes [7]. In our study, the significantly decreased SDNN, RMSDD, and PN50 values in children with TCA intoxication indicated the effect of TCAs on the cardiac autonomic system. Also, the negative correlation between TCA levels and SDNN values suggested that time-domain measures can be used as a marker to determine drug levels in cases of TCA intoxication. Decreased time-domain parameters, particularly SDNN, gave an idea about the association between the HRV results and ECG findings. Bjelakovic et al. [33] found a marked decrease in the SDNN and SDANN levels in adults with exercise-induced ventricular arrhythmia, and they suggested that this decrease might be related to the direct effect of the sympathetic system on myocardial electrical activity. Changes in the RMSDD and pNN50 components also indicated that the autonomic system was affected by TCA poisoning via the vagus nerve. 

The spectral analysis, including the short-term HRV recordings ranging between 2 to 5 minutes, is accepted as a noninvasive marker for the sympathetic and parasympathetic systems. In this way, heart rate signals are separated by frequency and intensity. Among these frequency bands, the LH and HF and their ratio (LF/HF) are frequently used [7]. The HF is thought to be the main marker of parasympathetic activity, but the LF reflects both the sympathetic and parasympathetic activity. Numerous investigators have emphasized that an increased LF/HF ratio shows sympathetic activity dominance. We evaluated the VLF, nLF, nHF, and LF/HF ratio during the first and last 5 minutes of the 24-hour HRV recordings. The nLF, nHF, and LF/HF ratio recorded at the intensive care unit were higher during the first 5 minutes of HRV recording in patients with TCA intoxication. During the last 5 minutes at the end of 24-hour record, the nLF levels were similar, the nHF level was lower, and the LF/HF remained high. One of the main determinants of parasympathetic activity is the high-frequency component (HF); thus, parasympathetic activity continued to be depressed in our patients after 24 hours of intoxication. These results indicated that the first 24-hour period is critical in TCA intoxication and that the changes that occurred at the end of the 24 hours may be explained by treatment for and elimination of the drug. While there were no differences in the nonspectral analysis parameters between patients with and without convulsions, the nLF and the LF/HF ratio were higher and the nHF was lower in patients with seizures. Frequency analysis in the first 5 minutes (especially LF/HF ratio) could be predictive of seizures and be an indication for anticonvulsant treatment. More comprehensive new studies are needed to evaluate the predictive value of the LF/HF ratio at admission for different treatment modalities.

Eisenhofer et al. [34] performed an experimental study for evaluation of the effects of desipramine (member of TCAs) on sympathetic nerves and they showed that desipramine has exerted differential central and peripheral effects (reduced sympathetic nerve outflow and diminished norepinephrine uptake). In humans, while desipramine diminished to norepinephrine uptake, it increased cardiac norepinephrine spillover by 25% [35]. There have been publications relating to HRV changes in adults and children who received TCA therapeutically [19, 36–39]. Srinivasan et al. [39] reported a marked decrease in the HF and an increased LF/HF ratio in the standing position, indicating the dose effects of therapeutic TCA on the cardiac autonomic system in children. Regarding TCA intoxication, antidepressant drugs are among the most common means of attempting suicide, and they are a serious risk factor for arrhythmias. Numerous studies have been performed to determine the development, type, and the severity of arrhythmia, and no standard indicator has yet been found [20]. It has been shown that HRV is markedly suppressed after imipramine and amitriptyline intake, and even if the EGC is normal, there is an association between the development of ventricular arrhythmia and increased LF/HF ratios [21]. Waring et al. [20] compared the results of HRV analysis in patients with antidepressant drug intoxication, and they found markedly prolonged QT and QTc intervals in patients who had received antidepressants, with a slight decrease in the HF and a slight increase in the LF. Djonlagic et al. [21] suggested that HRV is useful to assess the autonomic status of the cardiac system during the early and late terms of TCA intoxication. In particular, the cardiac autonomic system status can be obtained using HRV, and the risk of severe arrhythmia can be described in high-risk patients after TCA intoxication. Djonalgic et al. [21] reported the results of HRV in a 28-year-old patient who was referred with signs of TCA intoxication. In this case, the QRS complexes were wider than 150 ms, and the QTc interval was prolonged. The total power and LF and HF values were markedly suppressed, and the HRV depression continued during the second day without pathology on ECG. A markedly increased LF/HF ratio was noted before the development of ventricular arrhythmia. The heart rate and rhythm were markedly under the control of the autonomic nervous system. Thus, increased sympathetic activity in our study could result in clinical signs, particularly increased heart rate. It can be observed that parasympathetic suppression disappears, sympathetic activity becomes dominant, heart rate increases, and serious tachycardia occurs during TCA intoxication.

Power spectral analysis of HRV has been used frequently to assess cardiac autonomic function in different clinical condition; however, Goldstein et al. [40] highlighted that the relationship of LF power of HRV to cardiac sympathetic tone has been unclear. Manipulations and drugs that change LF/HF may do so not by affecting cardiac autonomic outflows directly but by affecting modulation of those outflows by baroreflexes. Watson et al. [41] showed that the increased cardiac sympathetic nerve activity is not dependent on depressed arterial baroreflex control of cardiac sympathetic nerve activity in experimental study about heart failure. For example, heart failure is characterized by activation of the sympathetic nervous system as demonstrated in patients by increases in circulating norepinephrine, total and regional norepinephrine spillover, and muscle sympathetic nerve activity. For this reason other determinant affecting HRV analysis including underlying disease, interventions, and drugs should be extensively evaluated during this analysis. Kingwell et al. [42] showed that the combination of the cardiac norepinephrine spillover technique and HRV will allow a more comprehensive assessment of both neuronal and postsynaptic aspects of the cardiac neuroeffector response. Our study, like previous clinical HRV studies, demonstrated some HRV abnormalities; however, extensive evaluation of cardiac autonomic activity needs includes other potential contributors.

In conclusion, autonomic changes that affect the heart rate may particularly cause ECG changes and arrhythmias that may develop in cases with TCA intoxication. Thus, treatments and antidotes the mechanisms of cause an increase in vagal activity may be effective in TCA intoxication. Short-term heart rate variability recordings may be a guide, especially for rhythm changes and as a predictor for seizures. We concluded that HRV could be used in the future as a noninvasive evaluation method for early diagnosis, risk determination, and determination of the effectiveness of treatment.

## Figures and Tables

**Table 1:** Clinical and laboratory findings of children with TCA intoxication.

Children with TCA intoxication (*n* = 20)	
Age	3–16 years
Gender	9 boys, 11 girls
TCA ingestion	Purpose of suicide (*n* = 16)
	Accidently (*n* = 4)
Serum TCA levels	1116 ± 635 g/mL
	(380–2000 ng/mL)
Loss of consciousness	16/20
Seizures	7/20
Median heart rate	148 (66–224)
Median systolic blood pressure (mmHg)	115 (62–160)
Median diastolic blood pressure (mmHg)	58 (36–92)
Abnormal ECG results	12 (20)
Prolonged PR interval	3 cases (0.24, 0.28, 0.24 second)
Prolonged QTc interval	5 cases (0.48, 0.52, 0.48, 0.47, 0.51)
Prolonged QRS interval	4 cases (>100 ms for all 4 cases)

**Table 2:** Twenty-four-hour time-domain (non-spectral) and 5-minute spectral analysis results by study group.

	TCA intoxication (*n* = 20)	Control (*n* = 20)	*P*
Mean heart rate (per minute)	148 (66–224)	78 (55–102)	**<0.05**
SDNN (ms)	104 (94–117)	146 (134–170)	**0.000**
SDANN (ms)	27 (25–44)	31 (26–39)	*P* > 0.05
RMSDD (ms)	46 (41–64)	65 (57–102)	**0.049**
SDNN index	60 (51–70)	81 (72–96)	**0.002**
pNN50 (%)	11.3 (8.9–20.9)	24.6 (20.8–33.4)	**0.005**

	Frequency-domain (spectral) analysis		

VLF (ms^2^)	772 (271–3873)	806 (698–1046)	*P* > 0.05
nLF (nu)	27 (24–35)	17 (11–24)	**0.012**
nHF (nu)	6.9 (5.4–8.7)	12.1 (10.7–22)	**0.001**
LF/HF	4.6 (1.1–14.4)	0.96 (0.8–2)	**0.001**

*Values are given as median (95% CI).

## References

[1] Litovitz TL, Klein-Schwartz W, Caravati EM, Youniss J, Crouch B, Lee S (1999). 1998 annual report of the American Association of Poison Control Centers Toxic Exposure Surveillance System. *American Journal of Emergency Medicine*.

[2] Andıran N, Sarıkayalar F (2004). Pattern of acute poisonings in children in Ankara: what has changed in twenty years?. *The Turkish Journal of Pediatrics*.

[3] Gillman PK (2007). Tricyclic antidepressant pharmacology and therapeutic drug interactions updated. *British Journal of Pharmacology*.

[4] Kerr GW, McGuffie AC, Wilkie S (2001). Tricyclic antidepressant overdose: a review. *Emergency Medicine Journal*.

[5] Caravati EM (1999). The electrocardiogram as a diagnostic discriminator for acute tricyclic antidepressant poisoning. *Journal of Toxicologyy*.

[6] Boehnert MT, Lovejoy FH (1985). Value of the QRS duration versus the serum drug level in predicting seizures and ventricular arrhythmias after an acute overdose of tricyclic antidepressants. *The New England Journal of Medicine*.

[7] Task Force of the European Society of Cardiology and the North American Society of Pacing and Electrophysiology (1996). Heart rate variability: standards of measurement, physiological interpretation, and clinical use. *European Heart Journal*.

[8] Vanderlei LCM, Pastre CM, Hoshi RA, de Carvalho TD, de Godoy MF (2009). Basic notions of heart rate variability and its clinical applicability. *Brazilian Journal of Cardiovascular Surgery*.

[9] Kaufman CL, Kaiser DR, Steinberger J, Kelly AS, Dengel DR (2007). Relationships of cardiac autonomie function with metabolic abnormalities in childhood obesity. *Obesity*.

[10] Rodríguez-Colón SM, Bixler EO, Li X, Vgontzas AN, Liao D (2011). Obesity is associated with impaired cardiac autonomic modulation in children. *International Journal of Pediatric Obesity*.

[11] Rutjanaprom W, Kanlop N, Charoenkwan P (2009). Heart rate variability in beta-thalassemia patients. *European Journal of Haematology*.

[12] Kardelen F, Tezcan G, Akcurin G, Ertug H, Yesilipek A (2008). Heart rate variability in patients with thalassemia major. *Pediatric Cardiology*.

[13] Hallioglu O, Okuyaz C, Mert E, Makharoblidze K (2008). Effects of antiepileptic drug therapy on heart rate variability in children with epilepsy. *Epilepsy Research*.

[14] Akçaboy M, Atalay S, Uçar T, Tutar E (2011). Heart rate variability during asymptomatic periods in children with recurrent neurocardiogenic syncope. *The Turkish Journal of Pediatrics*.

[15] Kwok KL, Yung TC, Ng DK, Chan CH, Lau WF, Fu YM (2011). Heart rate variability in childhood obstructive sleep apnea. *Pediatric Pulmonology*.

[16] Muzumdar HV, Sin S, Nikova M, Gates G, Kim D, Arens R (2011). Changes in heart rate variability after adenotonsillectomy in children with obstructive sleep apnea. *Chest*.

[17] Huikuri HV, Raatikainen MJP, Moerch-Joergensen R (2009). Prediction of fatal or near-fatal cardiac arrhythmia events in patients with depressed left ventricular function after an acute myocardial infarction. *European Heart Journal*.

[18] La Rovere MT, Pinna GD, Maestri R (2003). Short-term heart rate variability strongly predicts sudden cadiac death in chronic heart failure patients. *Circulation*.

[19] Kemp AH, Quintana DS, Gray MA, Felmingham KL, Brown K, Gatt JM (2010). Impact of depression and antidepressant treatment on heart rate variability: a review and meta-analysis. *Biological Psychiatry*.

[20] Waring WS, Rhee JY, Bateman DN, Leggett GE, Jamie H (2008). Impaired heart rate variability and altered cardiac sympathovagal balance after antidepressant overdose. *European Journal of Clinical Pharmacology*.

[21] Djonlagic I, Djonlagic H, Kibbel T, Suefke S, Dodt C (2007). Heart rate variability reveals risk of arrhythmias after intoxication with antidepressants. *Intensive Care Medicine*.

[22] Manikoth P, Subramanyan R, Menon S, Al Khusaiby SM (1999). A child with cardiac arrhythmia and convulsions. *The Lancet*.

[23] Rosenbaum TG, Kou M, Love JN (2005). Are one or two dangerous? Tricyclic antidepressant exposure in toddlers. *Journal of Emergency Medicine*.

[24] Leonard HL, Meyer MC, Swedo SE (1995). Electrocardiographic changes during desipramine and clomipramine treatment in children and adolescents. *Journal of the American Academy of Child and Adolescent Psychiatry*.

[25] Sarko J (2000). Antidepressants, old and new: a review of their adverse effects and toxicity in overdose. *Emergency Medicine Clinics of North America*.

[26] Çitak A, Soysal DD, Üçsel R, Karaböcüoglu M, Uzel N (2006). Seizures associated with poisoning in children: tricyclic antidepressant intoxication. *Pediatrics International*.

[27] James LP, Kearns GL (1995). Cyclic antidepressant toxicity in children and adolescents. *Journal of Clinical Pharmacology*.

[28] Palatini P, Julius S (2009). The role of cardiac autonomic function in hypertension and cardiovascular disease. *Current Hypertension Reports*.

[29] Haensel A, Mills PJ, Nelesen RA, Ziegler MG, Dimsdale JE (2008). The relationship between heart rate variability and inflammatory markers in cardiovascular diseases. *Psychoneuroendocrinology*.

[30] Anderson KP (2003). Sympathetic nervous system activity and ventricular tachyarrhythmias: recent advances. *Annals of Noninvasive Electrocardiology*.

[31] Algra A, Tijssen JGP, Roelandt JRTC, Pool J, Lubsen J (1993). Heart rate variability from 24-hour electrocardiography and the 2-year risk for sudden death. *Circulation*.

[32] Folino AF, Russo G, Bauce B, Mazzotti E, Daliento L (2004). Autonomic profile and arrhythmic risk stratification after surgical repair of tetralogy of Fallot. *American Heart Journal*.

[33] Bjelakovic B, Ilic S, Chouliaras K (2010). Heart rate variability in children with exercise-induced idiopathic ventricular arrhythmias. *Pediatric Cardiology*.

[34] Eisenhofer G, Saigusa T, Esler MD, Cox HS, Angus JA, Dorward PK (1991). Central sympathoinhibition and peripheral neuronal uptake blockade after desipramine in rabbits. *American Journal of Physiology*.

[35] Esler MD, Wallin G, Dorward PK (1991). Effects of desipramine on sympathetic nerve firing and norepinephrine spillover to plasma in humans. *American Journal of Physiology*.

[36] Udupa K, Sathyaprabha TN, Thirthalli J (2007). Alteration of cardiac autonomic functions in patients with major depression: a study using heart rate variability measures. *Journal of Affective Disorders*.

[37] Lederbogen F, Gernoth C, Weber B (2001). Antidepressive treatment with amitriptyline and paroxetine: comparable effects on heart rate variability. *Journal of Clinical Psychopharmacology*.

[38] Arranto CA, Mueller C, Hunziker PR, Maysch SC, Eriksson U (2003). Adverse cardiac events in ICU patients with presumptive antidepressant overdose. *Swiss Medical Weekly*.

[39] Srinivasan K, Ashok MV, Vaz M, Yeragani VK (2004). Effect of imipramine on linear and nonlinear measures of heart rate variability in children. *Pediatric Cardiology*.

[40] Goldstein DS, Bentho O, Park MY, Sharabi Y (2011). Low-frequency power of heart rate variability is not a measure of cardiac sympathetic tone but may be a measure of modulation of cardiac autonomic outflows by baroreflexes. *Experimental Physiology*.

[41] Watson AMD, Hood SG, Ramchandra R, McAllen RM, May CN (2007). Increased cardiac sympathetic nerve activity in heart failure is not due to desensitization of the arterial baroreflex. *American Journal of Physiology*.

[42] Kingwell BA, Thompson JM, Kaye DM, McPherson GA, Jennings GL, Esler MD (1994). Heart rate spectral analysis, cardiac norepinephrine spillover, and muscle sympathetic nerve activity during human sympathetic nervous activation and failure. *Circulation*.

